# Nonlinear relationship between atherogenic index of plasma and the risk of prediabetes: a retrospective study based on Chinese adults

**DOI:** 10.1186/s12933-023-01934-0

**Published:** 2023-08-10

**Authors:** Xiaodan Zheng, Xin Zhang, Yong Han, Haofei Hu, Changchun Cao

**Affiliations:** 1 Department of Neurology, Shenzhen Samii Medical Center (The Fourth People’s Hospital of Shenzhen), Shenzhen, Guangdong Province 518000 China; 2 Department of Rehabilitation, Shenzhen Dapeng New District Nan’ao People’s Hospital, No. 6, Renmin Road, Dapeng New District, Shenzhen, Guangdong Province 518000 China; 3 Department of Emergency, Shenzhen Second People’s Hospital, The First Affiliated Hospital of Shenzhen University, Shenzhen, Guangdong 518000 China; 4 Department of Nephrology, Shenzhen Second People’s Hospital, The First Affiliated Hospital of Shenzhen University, Shenzhen, Guangdong 518000 China; 5 Department of Emergency, Shenzhen Second People’s Hospital, The First Affiliated Hospital of Shenzhen University, No.3002, Sungang West Road, Futian District, Shenzhen, Guangdong Province 518000 China; 6 Department of Nephrology, Shenzhen Second People’s Hospital, The First Affiliated Hospital of Shenzhen University, No.3002, Sungang West Road, Futian District, Shenzhen, Guangdong Province 518000 China

**Keywords:** Atherogenic index of plasma, Prediabetes, Non-linearity

## Abstract

**Background:**

The atherogenic index of plasma (AIP) can reflect the burden of atherosclerosis. Hyperglycemia is one of the leading causes of atherosclerosis. However, the relationship between AIP and prediabetes is rarely studied. Therefore, we aimed to explore the relationship between AIP and prediabetes.

**Methods:**

This retrospective cohort study recruited 100,069 Chinese adults at the Rich Healthcare Group from 2010 to 2016. AIP was calculated according to Log10 (triglyceride/high-density lipoprotein cholesterol) formula. Cox regression method, sensitivity analyses and subgroup analyses were used to examine the relationship between AIP and prediabetes. Cox proportional hazards regression with cubic spline functions and smooth curve fitting was performed to explore the non-linearity between AIP and prediabetes. The two-piece Cox proportional hazards regression model was used to determine the inflection point of AIP on the risk of prediabetes.

**Results:**

After adjusting for confounding covariates, AIP was positively associated with prediabetes (HR: 1.41, 95%CI: 1.31–1.52, P < 0.0001). The two-piecewise Cox proportional hazards regression model discovered that the AIP’s inflection point was 0.03 (P for log-likelihood ratio test < 0.001). AIP was positively associated with the risk of prediabetes when AIP ≤ 0.03 (HR: 1.90, 95%CI: 1.66–2.16, P < 0.0001). In contrast, When AIP > 0.03, their association was not significant (HR: 1.04, 95%CI: 0.91–1.19, P = 0.5528).

**Conclusion:**

This study shows that AIP was positively and non-linearly associated with the risk of prediabetes after adjusting for other confounding factors. When AIP ≤ 0.03, AIP was positively associated with the risk of prediabetes.

**Supplementary Information:**

The online version contains supplementary material available at 10.1186/s12933-023-01934-0.

## Background

Prediabetes is defined as a condition in which blood glucose parameters are above normal but below the threshold for diabetes, and it is a high-risk state for developing diabetes [1]. The International Diabetes Federation reported that prevalence rates of prediabetes were 7.7% worldwide in 2017, which affected around 374 million people [2]. It is predicted by the International Diabetes Federation that 548 million adults will have prediabetes by 2045, representing 8.6% of adults worldwide [3]. Approximately 5–10% of adults with prediabetes develop diabetes each year, and about 70% of adults with prediabetes become diabetes finally [4]. Some research reported that individuals with prediabetes are at higher risk for cardiovascular disease, which indicates that the pathogenic effects of impaired glucose may begin even before people develop diabetes [5, 6]. The increasing prevalence of prediabetes worldwide and its complications make blood glucose disorder a serious public health problem. In order to prevent and treat diabetes in its early stages, many studies explored the risk factors for prediabetes and diabetes [7–10]. While prediabetes may represent a transient intermediary phase, its presence substantially heightens the likelihood of subsequent development of both type 2 diabetes and cardiovascular ailments [11, 12]. Furthermore, when patients with prediabetes have dyslipidemia, the risk of developing diabetes and cardiovascular disease is markedly amplified [13, 14]. Evidence from previous studies also pointed out that lifestyle changes, medication, and control of dyslipidemia can prevent prediabetes from developing into diabetes [15, 16]. Therefore, it is crucial to screen for risk factors for prediabetes and to treat these conditions as early as possible to prevent the disease from progressing and suffering negative effects.

Prediabetes is associated with a higher prevalence of dyslipidemia [17]. Similar to type 2 diabetes mellitus (T2DM), decreased high-density lipoprotein cholesterol (HDL-C), hypertriglyceridemia and increased small dense low-density lipoprotein (LDL) particles make up the characteristic pattern of dyslipidemia in prediabetes [18]. Although guidelines encourage intensive management of lipid parameters in individuals with diabetes, therapy of dyslipidemia in prediabetes is rarely mentioned [19]. Recently, it has been proposed that the atherogenic index of plasma (AIP), which is the logarithm of the ratio between the triglyceride (TG) and HDL-C in molar concentration, is connected to the burden of atherosclerosis [20]. Additionally, AIP can reflect the severity of insulin resistance, which is related to dysfunctional glucose metabolism [21]. Previous studies proposed that AIP was a useful lipid parameter to assess the risk of T2DM [22]. However, in the realm of academic literature, prior investigations concerning the association between TG/HDL-C and prediabetes have predominantly relied on cross-sectional study designs [23–25]. Evidence of evidence-based medicine is lower in cross-sectional studies compared to cohort studies. To fill the current research gap, our study aimed to quantitatively investigate the exact relationship between AIP and the risk of developing prediabetes in large Chinese subjects.

## Methods

### Data source

In the Dryad Digital Repository, researchers can download original data for free and cite them. We downloaded the raw data uploaded by Chen et al. [26]. Data including 211,833 Chinese people were downloaded from the Dryad data repository (dataset: https://datadryad.org/stash/dataset/doi:10.5061%2Fdryad.ft8750v). Under Dryad’s terms of service, researchers can use the data for secondary analysis. A secondary investigation of a medical examination program with public data was conducted in our study.

### Study population

The original study was approved by the Rich Healthcare Group Review Board. Hence, ethical approval was not required for this secondary analysis. Additionally, the initial study was completed according to principals of the Declaration of Helsinki. All procedures followed relevant guidelines and regulations.

**Fig. 1:**
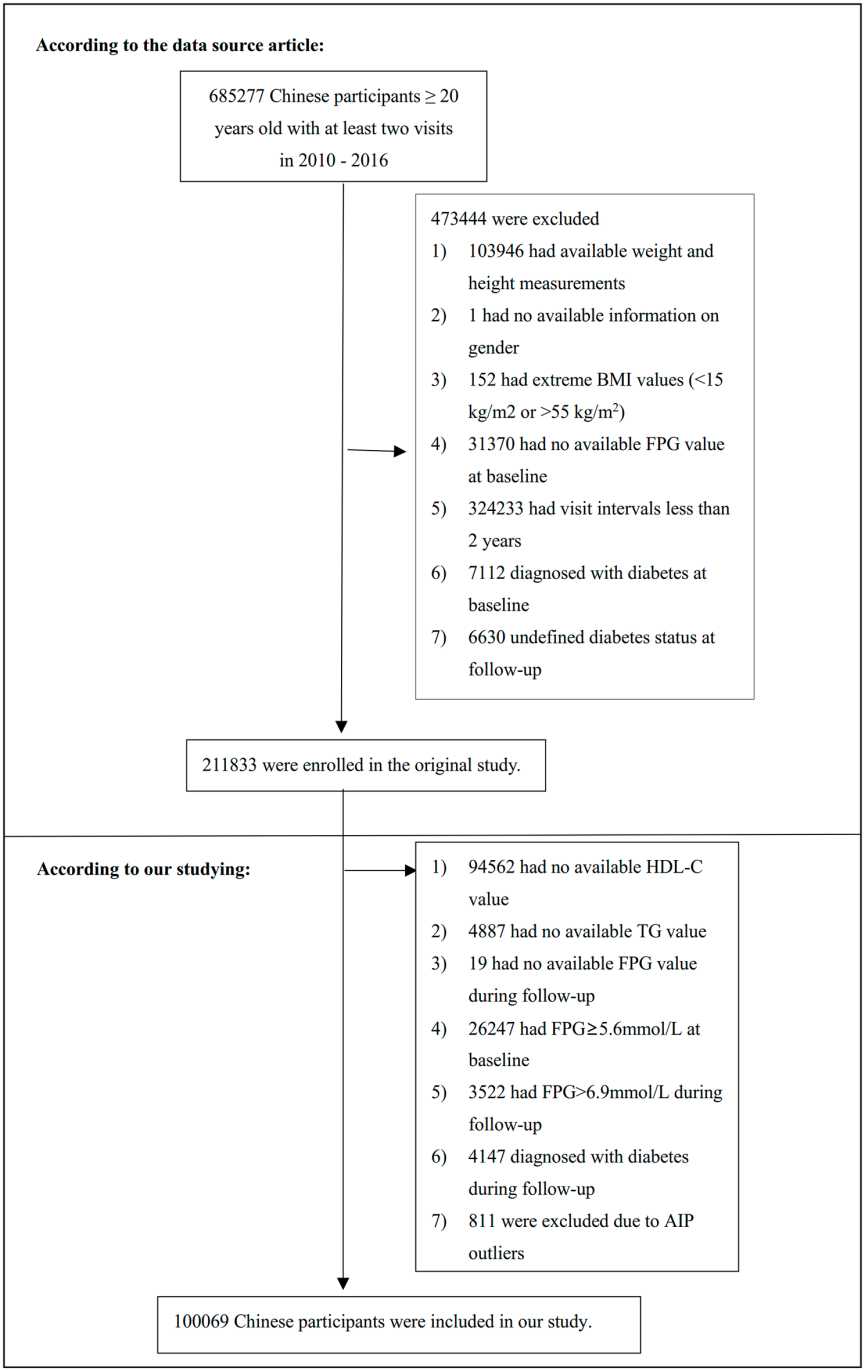
Study Population

The original study recruited 685,277 Chinese adults > 20 years old with at least two visits, covering 32 sites and 11 cities in China. Exclusion criteria were as follows: [1] diagnosis of diabetes at baseline and follow-up; [2] not defined diabetes status at follow-up; [3] abnormal body mass index (BMI), defined as a BMI > 55 or < 15 kg/m^2^; [4] missing data for weight, height, sex, HDL-C, TG, or fasting plasma glucose (FPG) at baseline, or FPG during follow-up; [5] had an FPG > 5.6mmol/L at baseline and an FPG > 6.9mmol/L during follow-up; [6] had a follow-up time of fewer than two years; [7] abnormal AIP (three standard deviations above or below the mean). Finally, 100,069 participants were finally included in the study. The study design and process were described in Fig. 1.

### Data collection

Trained staff collected and sorted the data. A standardized setting was used in the initial study to collect laboratory data and standardized practices were used to process the data. Demographic information was gathered by the skilled personnel, including age, systolic blood pressure (SBP), diastolic blood pressure (DBP), height and weight. Professional trainees measured individuals’ height and weight without light clothing and shoes. BMI was calculated in kg/m^2^ by dividing weight by height squared. Using a conventional mercury sphygmomanometer, trained staff members measure blood pressure. Additionally, the skilled team measured clinical data using a Beckman 5800 autoanalyzer, including FPG, HDL-C, TG, LDL cholesterol (LDL-C), total cholesterol (TC), blood urea nitrogen (BUN), serum creatinine (Scr), alanine aminotransferase (ALT) and aspartate aminotransferase (AST). Log10 (TG/HDL-C) was the formula used to calculate AIP in detail. The target independent was AIP at baseline, while the dependent variable was incident prediabetes during follow-up.

### Definition

The definition of prediabetes was impaired fasting glucose levels (FPG: 5.6–6.9 mmol/l).

### Statistical analysis

We performed statistical analyses by using R software version 3.6.1 (http://www.R-project.org/) and Empower Stats (R) version 2.2 (www.empowerstats.com, X&Y Solutions, Inc., Boston, MA).

AIP was divided into four groups based on quartiles: Q1 ≤ -0.299; -0.299 < Q2 ≤ -0.111; -0.111 < Q3 ≤ 0.098; Q4 > 0.098. Mean with standard deviation was used to represent continuous variables that followed a normal distribution, while median with interquartile range was used to represent continuous variables with skewed distribution. The percentages of various groups were used to represent categorical variables. One-way ANOVA or Kruskal-Wallis was performed to compare continuous variables and chi-square test was conducted to compare categorical variables. We used person-years and cumulative incidence rates to express incidence. Kaplan-Meier method was applied to compare survival and cumulative event rates. Using the log-rank test, we analyzed the Kaplan-Meier hazard ratios (HR) of adverse events.

Due to the excessive number of missing values for AST, smoking status, and drinking status, we first transformed the AST categorical variables based on tertiles. Then the missing values for smoking status, drinking status, and AST were treated as a separate group (i.e., Not recorded group). There were 13 (0.013%), 13 (0.013%), 162 (0.161%), 1 (0.001%), 2307 (2.289%), 1216 (1.205%), and 372 (0.369%) individuals with missing data for SBP, DBP, LDL-C, TC, BUN, Scr, and ALT, respectively. Our study used the interpolation model to deal with missing data for multiple variables, including age, gender, BMI, alcohol drinking status, smoking status, DBP, SBP, TC, LDL-C, AST, ALT, Scr, BUN, FPG and family history of diabetes. We used linear regression and 10 iterations to create the interpolation model. Analysis of missing data was based on the assumption of random missingness.

The effect of each variable on the risk of prediabetes was assessed using the univariate Cox regression method. The precise relationship between the AIP and the risk of prediabetes was also analyzed using the multivariate Cox regression analysis. Besides, we conducted non-adjusted model, minimally-adjusted model and fully-adjusted model to further study the relationship of AIP with the risk of developing prediabetes. When these covariances were added to the adjusted model, we only made adjustments for them if the HR varied by at least 10%.

A variety of sensitivity analyses were conducted to check whether the conclusions were reliable. Based on the quartile, AIP was converted into a categorical variable. The P for the trend was calculated in order to confirm the findings for AIP as the continuous variable and evaluate for nonlinearity. The elderly and obesity were connected with a higher incidence of prediabetes. To investigate the relationship between AIP and prediabetes risk, we excluded people with age ≥ 60 years or BMI ≥ 25 kg/m^2^ for further sensitivity analyses. A generalized additive model (GAM) was performed to test the validity of the results, which incorporated continuous variables as curves in the equation. We also calculated E-values to examine the possibility of unmeasured confounding between AIP and the risk of prediabetes [27].

We used Cox proportional hazards regression with cubic spline functions and smooth curve fitting to explore the nonlinear relationship between AIP and prediabetes. To address nonlinearity, our approach involves an academic methodology. Initially, we employ a recursive algorithm to determine the inflection point. The recursive algorithm commences with an arbitrary initialization and subsequently undergoes a series of filtering and smoothing steps in order to identify the inflection point accurately. Following this, we construct a two-piece Cox proportional hazards regression model, separately analyzing the data on either side of the inflection point. This rigorous analytical framework allows us to effectively account for and interpret the nonlinear relationship in the data. The log-likelihood ratio was used to identify the most suitable model for describing the link between AIP and prediabetes risk.

In order to analyze the subgroups (age, sex, family history of diabetes, BMI, SBP, DBP, drinking status and smoking status), the Cox proportional hazard model was also performed. According to the clinical cut point, age (< 60, ≥ 60 years), BMI (< 25, ≥ 25 kg/m^2^), SBP (< 140, ≥ 140 mmHg) and DBP (< 90, ≥ 90 mmHg) were converted into categorical variables. In addition to the stratification variables, every stratification was subjected to a fully adjusted analysis. We conducted a likelihood ratio test to confirm the interactions between subgroups. P values ≤ 0.05 were considered statistically significant.

## Results

### Baseline characteristics of participants

In the present study, 100,069 individuals without prediabetes at baseline were included. The average age was 42.90 ± 12.45 years and 51.86% of individuals were male. 12,292 individuals eventually got prediabetes after a follow-up of an average of 3.12 years. Table 1 displays fundamental indicators, laboratory tests, and other factors. AIP quartiles (Q1 ≤ -0.299; -0.299 < Q2 ≤ -0.111; -0.111 < Q3 ≤ 0.098; Q4 > 0.098) were used to divide the subjects into four groups. Compared to the other three groups, the Q4 group had higher age, SBP, DBP, BMI, AST, ALT, TG, LDL-C, TC, BUN, Scr and FPG. Additionally, there were more men, smokers and drinkers in the Q4 group. In comparison to the other three groups, the Q1 group had higher HDL-C. There was no significant difference in the proportion of family history of diabetes among the four groups.

**Table 1:** The Baseline Characteristics of Participants

AIP	Q1(≤-0.299)	Q2(-0.299 to ≤-0.111)	Q3(-0.111 to ≤ 0.098)	Q4(> 0.098)	P-value
Participants	25,017	25,016	25,016	25,020	
Gender					< 0.001
Male	6457 (25.81%)	11,221 (44.86%)	15,112 (60.41%)	19,101 (76.34%)	
Female	18,560 (74.19%)	13,795 (55.14%)	9904 (39.59%)	5919 (23.66%)	
Age(years)	39.46 ± 10.92	41.97 ± 12.34	44.26 ± 12.90	45.91 ± 12.61	< 0.001
Drinking status					
Current-drinker	63 (0.25%)	116 (0.46%)	187 (0.75%)	272 (1.09%)	
Ex- drinker	595 (2.38%)	1005 (4.02%)	1245 (4.98%)	1688 (6.75%)	
Never- drinker	5158 (20.62%)	5416 (21.65%)	5669 (22.66%)	6131 (24.50%)	
Not recorded	19,201 (76.75%)	18,479 (73.87%)	17,915 (71.61%)	16,929 (67.66%)	
Smoking status					< 0.001
Current-smoker	454 (7.81%)	920 (14.07%)	1483 (20.88%)	2451 (30.29%)	
Ex-smoker	110 (1.89%)	224 (3.43%)	338 (4.76%)	411 (5.08%)	
Never-smoker	5252 (20.99%)	5393 (21.56%)	5280 (21.11%)	5229 (20.90%)	
Not recorded	19,201 (76.75%)	18,479 (73.87%)	17,915 (71.61%)	16,929 (67.66%)	
Family history of diabetes					0.882
No	24,480 (97.85%)	24,466 (97.80%)	24,471 (97.82%)	24,457 (97.75%)	
Yes	537 (2.15%)	550 (2.20%)	545 (2.18%)	563 (2.25%)	
SBP (mmHg)	112.37 ± 14.46	116.27 ± 15.45	119.87 ± 16.08	123.68 ± 16.04	< 0.001
DBP (mmHg)	69.95 ± 9.74	72.38 ± 10.19	74.73 ± 10.63	77.86 ± 10.83	< 0.001
BMI (kg/m2)	21.22 ± 2.54	22.36 ± 2.88	23.62 ± 3.03	25.14 ± 2.99	< 0.001
AST					< 0.001
Low	4655 (18.61%)	3946 (15.77%)	3247 (12.98%)	1892 (7.56%)	
Medium	3425 (13.69%)	3700 (14.79%)	3703 (14.80%)	3148 (12.58%)	
High	2233 (8.93%)	3000 (11.99%)	3859 (15.43%)	5153 (20.60%)	
Not recorded	14,704 (58.78%)	14,370 (57.44%)	14,207 (56.79%)	14,827 (59.26%)	
ALT (U/L)	13.7 (10.8, 18.4)	15.9 (12.0, 22.3)	19.0 (13.9, 27.2)	25.4 (17.9, 37.9)	< 0.001
HDL-C (mmol/L)	1.62 ± 0.30	1.44 ± 0.24	1.32 ± 0.23	1.15 ± 0.23	< 0.001
TG (mmol/L)	0.60 (0.50, 0.70)	0.90 (0.79, 1.01)	1.26 (1.10, 1.45)	2.10 (1.71, 2.66)	< 0.001
LDL-C (mmol/L)	2.54 ± 0.59	2.68 ± 0.63	2.83 ± 0.67	2.94 ± 0.72	
TC (mmol/L)	4.53 ± 0.79	4.62 ± 0.83	4.79 ± 0.88	5.04 ± 0.92	< 0.001
BUN (mmol/L)	4.55 ± 1.16	4.58 ± 1.18	4.67 ± 1.17	4.72 ± 1.12	< 0.001
Scr (umol/L)	63.46 ± 13.61	68.28 ± 15.76	72.08 ± 15.84	75.44 ± 14.91	
FPG (mmol/L)	4.72 ± 0.46	4.78 ± 0.46	4.81 ± 0.48	4.85 ± 0.47	< 0.001
AIP	-0.44 ± 0.11	-0.20 ± 0.05	-0.01 ± 0.06	0.29 ± 0.15	< 0.001

Values are n (%) or mean ± standard deviation

AIP: atherogenic index of plasma, SBP systolic blood pressure, DBP diastolic blood pressure, BMI body mass index, ALT alanine aminotransferase, AST aspartate aminotransferase, HDL-C high-density lipoprotein cholesterol, LDL-C low-density lipoprotein cholesterol, TC total cholesterol, TG triglycerides, Scr serum creatinine, BUN blood urea nitrogen, FPG fasting plasma glucose

### The incidence rate of prediabetes

Table 2 displays the incidence rate of prediabetes in 100,069 individuals over the duration of follow-up. In total, all people had an incidence rate of 12.28% (12.08-12.49%). The four AIP groups’ incidence rates were respectively 7.33% (7.01-7.65%), 10.36% (9.98-10.74%), 13.67% (13.24-14.09%) and 17.78% (17.30-18.25%). In addition, the accumulative incidence rate of the overall population and four AIP groups were 3939.86 per 100,000 person-years, 2352.85 per 100,000 person-years, 3349.24 per 100,000 person-years, 4396.60 per 100,000 person-years, and 5636.26 per 100,000 person-years, respectively. In comparison to participants with lower AIP groups, those with higher AIP groups had a greater incidence and cumulative incidence rate of prediabetes (p for trend < 0.001).

**Table 2:** Incidence rate of prediabetes

AIP	Participants (n)	prediabetes events (n)	Cumulative incidence (95%CI) (%)	Per 100,000 person-year
Total	100,069	12,292	12.28 (12.08–12.49)	3939.86
Q1	25,017	1834	7.33 (7.01–7.65)	2352.85
Q2	25,016	2591	10.36 (9.98–10.74)	3349.24
Q3	25,016	3419	13.67 (13.24–14.09)	4396.60
Q4	25,020	4448	17.78 (17.30-18.25)	5636.26
P for trend			< 0.001	< 0.001

**Fig. 2:**
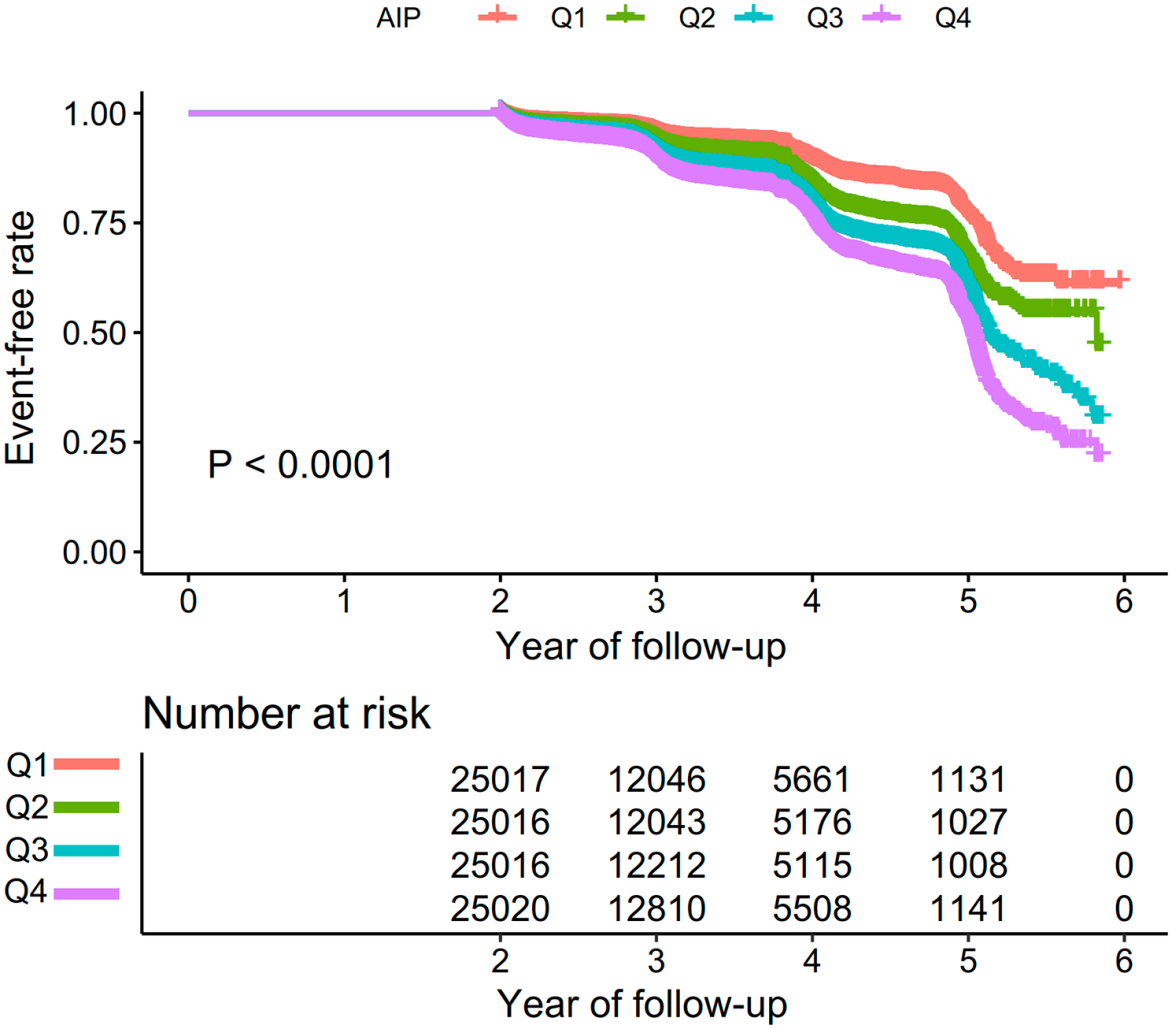
Kaplan–Meier event-free survival curve. Kaplan–Meier analysis of incident prediabetes based on AIP quartiles (log-rank, P < 0.0001)

As shown in Fig. 2, Kaplan-Meier curves indicated the likelihood of surviving without prediabetes. The risk of developing prediabetes was significantly different between the four AIP groups (P < 0.0001). As AIP levels increased, the likelihood of surviving without prediabetes gradually decreased. It indicated that the group with the highest AIP had the greatest risk of developing prediabetes.

### Univariate analysis

The results of the univariate analysis are presented in Table 3. The risk of prediabetes was positively associated with age, SBP, DBP, BMI, AST, ALT, TG, LDL-C, TC, BUN, Scr and FPG. There was a negative association between HDL-C and the risk of prediabetes. There has a lower risk of developing prediabetes in those who never drink or smoke. In comparison to men, women have a lower risk of developing prediabetes.

**Table 3:** The results of univariate analysis

	Statistics	HR (95%CI)	P value
Gender			< 0.0001
Male	51,891 (51.86%)	ref	
Female	48,178 (48.14%)	0.65 (0.63, 0.68)	< 0.0001
Age(years)	42.90 ± 12.45	1.03 (1.03, 1.03)	< 0.0001
Drinking status			
Current-drinker	638 (0.64%)	ref	
Ex-drinker	4533 (4.53%)	0.69 (0.56, 0.86)	0.0008
Never-drinker	22,374 (22.36%)	0.65 (0.53, 0.80)	< 0.0001
Not recorded	72,524 (72.47%)	0.72 (0.59, 0.88)	0.0015
Smoking status			
Current-smoker	5308 (5.30%)	ref	
Ex-smoker	1083 (1.08%)	0.86 (0.72, 1.02)	0.0763
Never-smoker	21,154 (21.14%)	0.71 (0.66, 0.77)	< 0.0001
Not recorded	72,524 (72.47%)	0.84 (0.79, 0.91)	< 0.0001
Family history of diabetes			0.7795
No	97,874 (97.81%)	ref	
Yes	2195 (2.19%)	0.98 (0.88, 1.10)	
SBP (mmHg)	118.05 ± 16.08	1.03 (1.02, 1.03)	< 0.0001
DBP (mmHg)	73.73 ± 10.76	1.03 (1.03, 1.03)	< 0.0001
BMI (kg/m2)	23.08 ± 3.22	1.12 (1.12, 1.13)	< 0.0001
AST			
Low	13,740 (13.73%)	ref	
Medium	13,976 (13.97%)	1.08 (1.01, 1.15)	0.0239
High	14,245 (14.24%)	1.36 (1.27, 1.44)	< 0.0001
Not recorded	58,108 (58.07%)	0.71 (0.67, 0.75)	< 0.0001
ALT (U/L)	23.02 ± 21.43	1.00 (1.00, 1.00)	< 0.0001
HDL-C (mmol/L)	1.38 ± 0.30	0.87 (0.82, 0.92)	< 0.0001
TG (mmol/L)	1.28 ± 0.82	1.36 (1.34, 1.38)	< 0.0001
LDL-C (mmol/L)	2.75 ± 0.67	1.27 (1.24, 1.30)	< 0.0001
TC (mmol/L)	4.75 ± 0.88	1.20 (1.18, 1.23)	< 0.0001
BUN (mmol/L)	4.63 ± 1.16	1.14 (1.12, 1.15)	< 0.0001
Scr (umol/L)	69.82 ± 15.70	1.01 (1.01, 1.01)	< 0.0001
FPG (mmol/L)	4.79 ± 0.47	5.57 (5.32, 5.83)	< 0.0001
AIP	-0.09 ± 0.28	2.83 (2.67, 3.00)	< 0.0001

### The relationship between AIP and prediabetes

The Cox proportional hazard regression models with the HR and 95% confidence interval (CI) for the association between AIP and prediabetes are presented in Table 4. The HR (95% CI) for prediabetes connection with AIP was 2.83 (2.67, 3.00) in the non-adjusted model. In the minimally-adjusted model with the adjustments for gender, age, SBP, DBP, family history of diabetes, drinking status, smoking status and BMI, the HR (95% CI) was 1.37 (1.28, 1.47). In the fully-adjusted model, after further adjusting for TC, LDL-C, AST, ALT, Scr, BUN and FPG, the HR (95% CI) was 1.41 (1.31, 1.52). This demonstrated that the risk of prediabetes increased by 41% for every unit increase in AIP.

**Table 4:** Relationship between AIP and incident prediabetes in different models

Variable	Non-adjusted model (HR.,95% CI, P)	Minimally-adjusted model (HR,95% CI, P)	Fully-adjusted model (HR,95% CI, P)	GAM (HR,95% CI, P)
AIP	2.83 (2.67, 3.00) < 0.0001	1.37 (1.28, 1.47) < 0.0001	1.41 (1.31, 1.52) < 0.0001	1.34 (1.24, 1.44) < 0.0001
AIP (quartile)				
Q1	ref	ref	ref	1.0
Q2	1.47 (1.39, 1.56) < 0.0001	1.19 (1.12, 1.26) < 0.0001	1.18 (1.11, 1.25) < 0.0001	1.15 (1.08, 1.23) < 0.0001
Q3	1.94 (1.83, 2.05) < 0.0001	1.29 (1.22, 1.37) < 0.0001	1.28 (1.20, 1.36) < 0.0001	1.23 (1.16, 1.31) < 0.0001
Q4	2.40 (2.27, 2.53) < 0.0001	1.33 (1.25, 1.42) < 0.0001	1.34 (1.26, 1.43) < 0.0001	1.28 (1.20, 1.37) < 0.0001
P for trend	< 0.0001	< 0.0001	< 0.0001	< 0.0001

Non-adjusted model: we did not adjust for other covariates

Minimally-adjusted model: we adjusted for gender, age, SBP, DBP, family history of diabetes, drinking status, smoking status, and BMI

Fully-adjusted model: we adjusted for gender, age, SBP, DBP, family history of diabetes, drinking status, smoking status, BMI, TC, LDL-C, AST, ALT, Scr, BUN and FPG

GAM: All covariates listed in Table 1 were adjusted. However, continuous covariates were adjusted as nonlinearity

HR, hazard ratios; CI, confidence interval; Ref, reference; GAM, generalized additive mode; AIP, atherogenic index of plasma

### The results of sensitivity analysis

To evaluate the robustness of our results, we further performed sensitivity analysis. AIP was converted from a continuous variable to a categorical variable, and it was then added back into the model after being categorically transformed. When transforming AIP into a categorical variable, the p for trend was not equal, suggesting a possible nonlinear association of AIP with prediabetes risk. As shown in Table 4, results from the GAM model were consistent with those from the fully adjusted model (HR: 1.34, 95%CI: 1.24–1.44). Additionally, an E-value was computed to assess the vulnerability of the study results to potential unobserved confounding factors. The resulting E-value (2.17) demonstrated a higher level of statistical significance in comparison to the relative risk (1.69) associated with unmeasured confounders and AIP. This suggests that the impact of unmeasured or unidentified confounders on the relationship between AIP and the occurrence of prediabetes was negligible.

Besides, we performed sensitivity analysis on individuals with a BMI < 25 kg/m^2^. There was also a positive relationship between AIP and prediabetes risk after adjusting for confounding covariates (HR: 1.51, 95%CI: 1.37–1.66) (Table 5). Individuals with age < 60 years were also included from other sensitivity analysis. The findings revealed that AIP was remained positively associated with the probability of developing prediabetes after controlling for confounding covariates (HR: 1.44, 95%CI: 1.32–1.56) (Table 5). According to the sensitivity analysis, it suggested that our results were well-robust.

**Table 5:** Relationship between AIP and prediabetes in different sensitivity analyses

Exposure	Model I (HR,95%CI, P)	Model II (HR,95%CI, P)
AIP	1.51 (1.37, 1.66) < 0.0001	1.44 (1.32, 1.56) < 0.0001
AIP (Quintile)		
Q1	ref	ref
Q2	1.15 (1.07, 1.23) < 0.0001	1.21 (1.13, 1.30) < 0.0001
Q3	1.23 (1.14, 1.32) < 0.0001	1.28 (1.20, 1.38) < 0.0001
Q4	1.32 (1.23, 1.43) < 0.0001	1.36 (1.27, 1.46) < 0.0001
P for trend	< 0.0001	< 0.0001

Model I was sensitivity analysis in participants with BMI < 25 kg/m^2^. We adjusted gender, age, SBP, DBP, family history of diabetes, drinking status, smoking status, TC, LDL-C, AST, ALT, Scr, BUN and FPG

Model II was sensitivity analysis in participants aged < 60 years. We adjusted gender, SBP, DBP, family history of diabetes, drinking status, smoking status, BMI, TC, LDL-C, AST, ALT, Scr, BUN and FPG

HR, hazard ratios; CI, confidence, Ref: reference; AIP: atherogenic index of plasma

### The nonlinear relationship between AIP and prediabetes

**Fig. 3:**
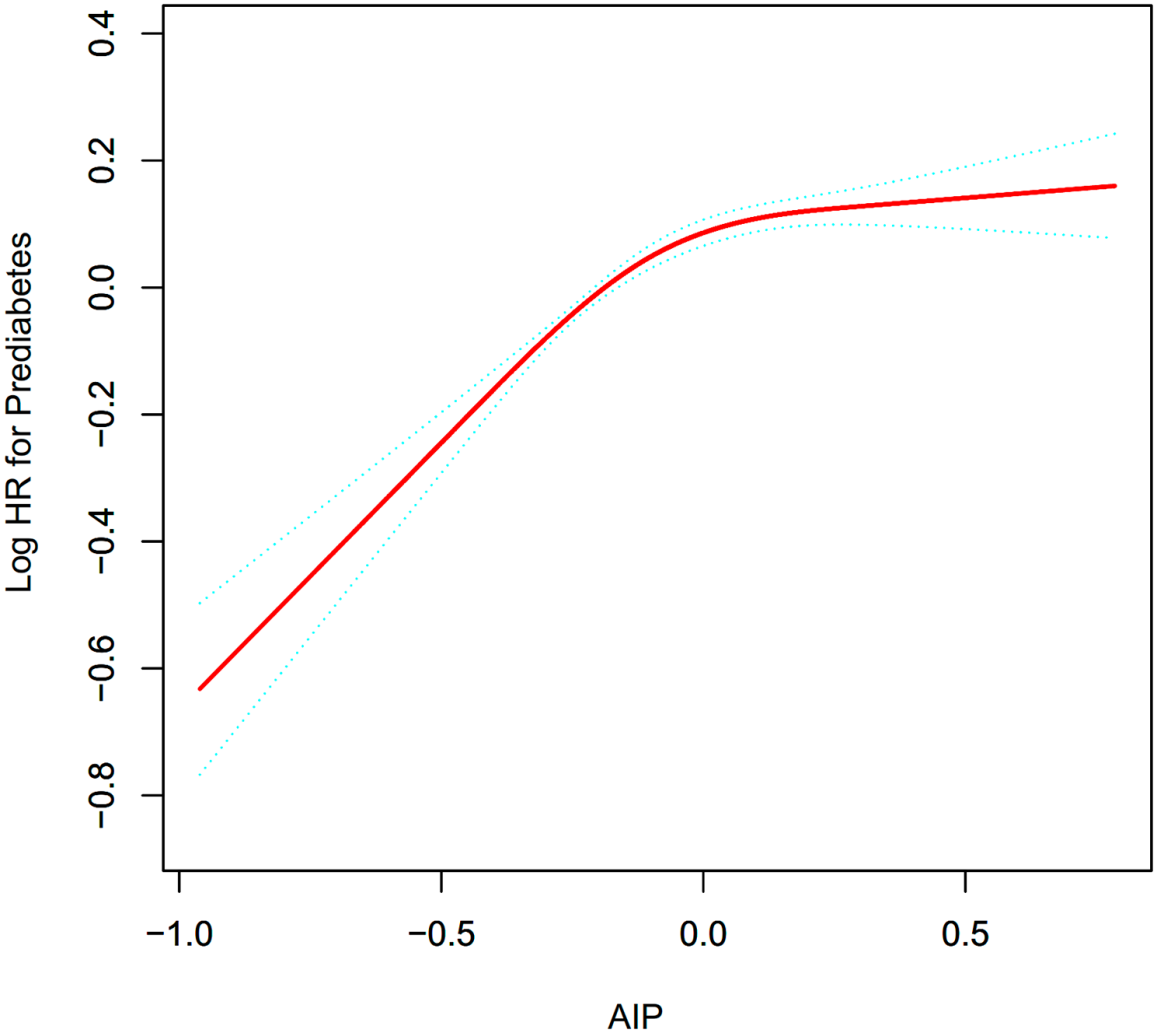
The nonlinear relationship between AIP and incident prediabetes. A nonlinear relationship between them was detected after adjusting for gender, age, SBP, DBP, family history of diabetes, drinking status, smoking status, BMI, TC, LDL-C, AST, ALT, Scr, BUN, FPG

Figure 3 displays Cox proportional hazards regression with cubic spline functions and smooth curve fitting to assess AIP’s non-linearity with prediabetes risk. After adjusting for confounding factors, the association between AIP and the probability of developing prediabetes was nonlinear (Table 6). The two-piecewise Cox proportional hazards regression model discovered that the AIP’s inflection point was 0.03 (P for log-likelihood ratio test < 0.001). When AIP ≤ 0.03, AIP was positively associated with the risk of prediabetes (HR: 1.90, 95%CI: 1.66–2.16, P < 0.0001). In contrast, When AIP > 0.03, their association was not significant (HR: 1.04, 95%CI: 0.91–1.19, P = 0.5528).

**Table 6:** The result of the two-piecewise Cox proportional hazards regression model

Incident prediabetes	HR (95%CI)	P
Fitting model by standard Cox proportional hazards regression	1.41 (1.31, 1.52)	< 0.0001
Fitting model by two-piecewise Cox proportional hazards regression		
Inflection points of AIP	0.03	
≤ 0.03	1.90 (1.66, 2.16)	< 0.0001
> 0.03	1.04 (0.91, 1.19)	0.5528
P for log likelihood ratio test	< 0.001	

We adjusted for gender, age, SBP, DBP, family history of diabetes, drinking status, smoking status, BMI, TC, LDL-C, AST, ALT, Scr, BUN, FPG

HR, hazard ratios; CI, confidence; AIP: atherogenic index of plasma

### Subgroup analysis

Additional risk factors that might have an impact on the relationship between AIP and prediabetes risk were explored by performing subgroup analysis. As stratification factors, we chose age, gender, smoking status, drinking status, family history of diabetes, SBP, DBP and BMI. We then examined trends in effect sizes for these factors (Table 7). Drinking status, smoking status, family history of diabetes, SBP and DBP had no impact on the association of AIP with prediabetes risk. The results revealed a stronger connection between AIP and prediabetes risk in people with age<60 years, females and individuals with SBP < 140mmHg and BMI < 25 kg/m^2^.

**Table 7:** Effect size of AIP on prediabetes in prespecified and exploratory subgroups

Characteristic	No of patients	HR (95%CI)	P value	P for interaction
Age(years)				< 0.0001
< 60	87,925	1.56 (1.43, 1.70)	< 0.0001	
≥ 60	12,144	1.11 (0.96, 1.29)	0.1670	
Gender				< 0.0001
Male	51,891	1.23 (1.12, 1.35)	< 0.0001	
Female	48,178	1.71 (1.52, 1.92)	< 0.0001	
Drinking status				0.0574
Current drinker	638	1.23 (0.53, 2.82)	0.6286	
Ever drinker	4533	1.52 (1.11, 2.09)	0.0089	
Never drinker	22,374	1.68 (1.45, 1.95)	< 0.0001	
Not recorded	72,524	1.34 (1.23, 1.45)	< 0.0001	
Smoking status				0.0828
Current-smoker	5308	1.59 (1.20, 2.09)	0.0010	
Ex-smoker	1083	1.30 (0.67, 2.50)	0.4402	
Never-smoker	21,154	1.69 (1.43, 1.99)	< 0.0001	
Not recorded	72,524	1.34 (1.23, 1.45)	< 0.0001	
Family history of diabetes				0.3600
No	97,874	1.41 (1.31, 1.51)	< 0.0001	
Yes	2195	1.72 (1.12, 2.64)	0.0128	
SBP (mmHg)				0.0389
< 140	91,009	1.43 (1.32, 1.55)	< 0.0001	
≥ 140	9060	1.18 (1.00, 1.39)	0.0550	
DBP (mmHg)				0.5907
< 90	92,459	1.41 (1.31, 1.53)	< 0.0001	
≥ 90	7610	1.34 (1.11, 1.61)	0.0025	
BMI (kg/m^2^)				< 0.0001
< 25	73,573	1.76 (1.61, 1.93)	< 0.0001	
≥ 25	26,496	1.23 (1.10, 1.37)	0.0002	

Note 1: Above model adjusted for gender, age, SBP, DBP, family history of diabetes, drinking status, smoking status, BMI, TC, LDL-C, AST, ALT, Scr, BUN, FPG

Note 2: In each case, the model is not adjusted for the stratification variable

## Discussion

Our retrospective study showed that higher AIP was associated with a higher risk of prediabetes. After adjusting for other covariates, the risk of prediabetes increased by 41% for every unit increase in AIP. On both the left and right side of the inflection point, it was found that the relationship between AIP and prediabetes was nonlinear. When AIP ≤ 0.03, AIP was positively associated with the risk of prediabetes. There was a stronger association between AIP and prediabetes risk in individuals aged < 60 years, women, or with SBP < 140mmHg and BMI < 25 kg/m^2^.

Previous studies also reported the association between dyslipidemia and prediabetes. TG/HDL-C were significantly correlated with prediabetes after adjusting for age, sex, blood pressure smoking status, BMI, FPG and 2-h post-challenge plasma glucose in a cross-sectional study enrolled 2680 participants (OR: 3.445, 95%CI: 2.417–4.921, P <0.001) [23]. A cross-sectional survey including 2293 adults in Rural Bangladeshi showed that prediabetes had a significant association with high TG (OR: 1.96, p < 0.001) and low HDL-C (OR: 2.93, p = 0.011) [24]. Another cross-sectional study involving 153,163 non-obese participants with a normal range of LDL-C found a positive relationship between TG/HDL-C and prediabetes after adjusting for confounding factors (OR:1.185, 95%CI: 1.145–1.226) [25]. These studies indicated that the trend of prediabetes is consistent with diabetes. Compared to other studies, our study provides new perspectives on the relationship between AIP and prediabetes. First, to the best of our knowledge, previous findings were from cross-sectional studies, but were not reported in cohort studies. Therefore, this cohort study aimed to gain insight into the relationship between AIP and prediabetes in a Chinese adult population. Second, the resolution of nonlinearity is a significant improvement compared with previous studies, which informs the management of AIP in Chinese adults. In addition, we controlled for more biochemical indicators in our study, such as Scr, AST, ALT, and family history of diabetes [28, 29]. There is evidence that these parameters are associated with prediabetes risk. In testing the robustness of the results through a series of sensitivity analyses (target independent variable transformation, subgroup analysis, and insertion of continuous covariates as curves into the equation using GAM), stronger positive correlations were found in women and in those aged < 60 years, women, or with SBP < 140mmHg and BMI < 25 kg/m^2^, which will be of clinical interest. Third, the above studies confirm that the relationship between AIP and risk of prediabetes is stable. More importantly, our findings provide a reference for clinical intervention of AIP levels to reduce the risk of prediabetes. Early intervention may improve prognosis if more lifestyle or therapeutic measures are taken to reduce AIP at an early stage.

Our study shows that the relationship between AIP and prediabetes is nonlinear after controlling for age, sex, SBP, DBP, family history of diabetes, drinking, smoking, BMI, TC, LDL-C, AST, ALT, Scr, BUN and FPG. Based on the two-piecewise Cox proportional hazards regression model, the AIP inflection point is calculated. When the AIP level is below 0.03, the risk of developing prediabetes increases by 90% for every unit increase in AIP level (HR: 1.90, 95%CI: 1.66–2.16, P < 0.0001). However, the AIP level is not related with incident prediabetes when the AIP level is above 0.03 (HR: 1.04, 95%CI: 0.91–1.19, P = 0.5528). The risk of developing prediabetes can be predicted based on the AIP values, which will alert participants to make early changes in lifestyle habits to reduce risk.

We speculated that there were possible mechanisms underlying the association of AIP with prediabetes. A higher concentration of TG contributed to the development of prediabetes primarily through free fatty acids [30]. By increasing free fatty acids, the formation of toxic lipids was increased, resulting in the alterations in insulin signaling of pancreatic α-cell and excessive secretion of glucagon [30]. Elevated glucagon levels were considered to be a significant factor in hyperglycemia [31]. Plasma glucagon promoted glycogenolysis and gluconeogenesis to stimulate the output of hepatic glucose [32]. In addition, a lower level of HDL-C could decrease cholesterol efflux, which resulted in cholesterol accumulation in the pancreatic β-cells and further caused β-cell dysfunction with impaired insulin secretion, elevated blood glucose and β-cell apoptosis [33–35]. These potential mechanisms contributed to give a pathophysiological explanation for the association between AIP and the development of prediabetes.

Our study has several following advantages. First, we further explored the nonlinear association between AIP and prediabetes. Second, residual confounding factors were minimized by using strict statistical adjustments. Third, sensitivity analyses were performed to ensure the robustness of the results. It included transforming AIP into a categorical variable, using GAM to insert the continuity covariate as a curve into the equation and calculating E-values to explore the potential for unmeasured confounding. The relationship between AIP and prediabetes was reanalyzed after excluding individuals with BMI ≥ 25 kg/m^2^ or age ≥ 60 years. Fourth, the present study conducted subgroup analysis to assess confounding factors that may influence the connection between AIP and prediabetes.

There are some limitations in our study. First, prediabetes may have been underestimated due to a lack of experimental OGTT. Second, the database lacks information on atherosclerosis, lipid-regulating medication and the presence or absence of hyperlipidemia, so we cannot perform a sub-analysis based on the presence or absence of hyperlipidemia or the use of lipid-regulating drugs. Third, similar to all observational studies, despite the control of known potential confounding factors such as BMI, TC, LDL-C, AST, ALT, Scr, BUN, and FPG, the presence of uncontrolled or unmeasured confounders, including diet and exercise, cannot be entirely ruled out. Nevertheless, we employed the E-value to assess the influence of unmeasured confounders and found it improbable that they accounted for the outcomes. In subsequent research, it would be advantageous to contemplate the incorporation of a comprehensive range of variables, encompassing data on diet and exercise, by either refining the study design or collaborating with other researchers. Fourth, TG and HDL-C were measured only at baseline in the original study. The initial study didn’t cover how TG and HDL-C fluctuated over time. Future designs of our investigation may include capturing additional confounding variables, such as variations in TG and HDL-C during follow-up. As a result, we might use a GAM model to investigate how changes in the AIP would affect future prediabetes risk.

## Conclusion

This study shows that AIP was positively and non-linearly associated with the risk of prediabetes after adjusting for other confounding factors. When AIP ≤ 0.03, AIP was positively associated with the risk of prediabetes.

### Electronic supplementary material

Below is the link to the electronic supplementary material.

Supplementary Material 1

## Data Availability

The raw data can be downloaded from the ‘DATADRYAD’ database (www.Datadryad.org). Dryad Digital Repository. https://datadryad.org/stash/dataset/doi:10.5061%2Fdryad.ft8750v.
