# Supplementary material for: Nonlinear relationship between atherogenic index of plasma and the risk of prediabetes: a retrospective study based on Chinese adults
Source: Cardiovasc Diabetol. 2023 Aug 10;22:205. doi: 10.1186/s12933-023-01934-0 (PMC10416492; doi:10.1186/s12933-023-01934-0)
Supplement: Supplementary file 1 — Supplementary Material 1 [file 12933_2023_1934_MOESM1_ESM.docx]

**Table S1** Relationship between AIP and prediabetes in different models

| Variable | Non-adjusted model (HR.,95% CI, P) | Minimally-adjusted model (HR,95% CI, P) | Fully-adjusted model (HR,95% CI, P) |
| --- | --- | --- | --- |
| AIP | 2.83 (2.67, 3.00) <0.0001 | 1.50 (1.40, 1.61) <0.0001 | 1.30 (1.21, 1.39) <0.0001 |
| AIP (quartile) |  |  |  |
| Q1 | ref | ref | ref |
| Q2 | 1.47 (1.39, 1.56) <0.0001 | 1.24 (1.17, 1.32) <0.0001 | 1.15 (1.08, 1.22) <0.0001 |
| Q3 | 1.94 (1.83, 2.05) <0.0001 | 1.40 (1.31, 1.48) <0.0001 | 1.27 (1.20, 1.35) <0.0001 |
| Q4 | 2.40 (2.27, 2.53) <0.0001 | 1.45 (1.36, 1.54) <0.0001 | 1.27 (1.19, 1.35) <0.0001 |
| P for trend | <0.0001 | <0.0001 | <0.0001 |

Non-adjusted model: we did not adjust for other covariates

Minimally-adjusted model: we adjusted for gender, age, SBP, DBP, family history of diabetes, and BMI.

Fully-adjusted model: we adjusted for gender, age, SBP, DBP, family history of diabetes, BMI, TC, LDL-C, ALT, Scr, BUN and FPG.

**Table S2** Relationship between AIP and prediabetes in different models

| Variable | Non-adjusted model (HR.,95% CI, P) | Minimally-adjusted model (HR,95% CI, P) | Fully-adjusted model (HR,95% CI, P) |
| --- | --- | --- | --- |
| AIP | 3.24 (2.70, 3.89) <0.0001 | 1.72 (1.38, 2.14) <0.0001 | 1.33 (1.06, 1.67) 0.0135 |
| AIP (quartile) |  |  |  |
| Q1 | ref | ref | ref |
| Q2 | 1.29 (1.05, 1.59) 0.0136 | 1.09 (0.89, 1.35) 0.3936 | 0.99 (0.80, 1.22) 0.9166 |
| Q3 | 1.84 (1.53, 2.22) <0.0001 | 1.32 (1.09, 1.61) 0.0053 | 1.18 (0.97, 1.44) 0.1073 |
| Q4 | 2.49 (2.09, 2.97) <0.0001 | 1.47 (1.20, 1.79) 0.0002 | 1.17 (0.95, 1.43) 0.1339 |
| P for trend | <0.0001 | <0.0001 | 0.0491 |

Non-adjusted model: we did not adjust for other covariates

Minimally-adjusted model: we adjusted for gender, age, SBP, DBP, family history of diabetes, drinking status, smoking status, and BMI.

Fully-adjusted model: we adjusted for gender, age, SBP, DBP, family history of diabetes, drinking status, smoking status, BMI, TC, LDL-C, AST, ALT, Scr, BUN and FPG.

Note: We excluded populations with missing data on smoking status, drinking status, and AST.
